# An Overview of Infant Mortality Trends in Qatar from 2004 to 2014

**DOI:** 10.7759/cureus.1669

**Published:** 2017-09-09

**Authors:** Mohammed Al-Thani, Al-Anoud Al-Thani, Amine Toumi, Shams Eldin Khalifa, Hammad Akram

**Affiliations:** 1 Ministry of Public Health, State of Qatar; 2 Health Intelligence and Information Section, Ministry of Public Health

**Keywords:** infant mortality, qatar, infant mortality rate, im causes, epidemiology, mortality trends

## Abstract

Background

Infant mortality is an important health indicator that estimates population well-being. Infant mortality has declined globally but is still a major public health challenge. This article provides the characteristics, causes, burden, and trends of infant mortality in Qatar.

Methods

Frequencies, percentages, and rates were calculated using data from birth-death registries over 2004–2014 to describe infant mortality by nationality, gender, and age group. We calculated the relative risks of the top causes of infant mortality among subgroups according to the 10^th^ Revision of the International Statistical Classification of Diseases and Related Health Problems (ICD-10, Version 2016).

Results

During 2004–2014, 204,224 live births and 1,505 infant deaths were recorded. The infant mortality rate (IMR) averaged 7.4/1000 live births (males 8.1, females 6.6, non-Qataris 7.7, and Qataris 6.8). IMR declined 20% from 2004 to 2014. The decline in IMR was significant for the overall population of infants (p=0.006), male infants (p=0.04), females (p=0.006), and for non-Qatari males (p=0.007) and non-Qatari females (p=0.007). The leading causes of infant mortality were congenital malformations (all types) (34.5%), low birth weight (LBW) (27%), and respiratory distress of newborns (2.8%). Male infants had a higher risk of mortality than female infants due to a congenital malformation of lungs (p=0.02), other congenital malformations, not elsewhere classified (p=0.01), and cardiovascular disorders (p=0.05).

Conclusion

The study shows that infant mortality among male infants is high due to the top infant mortality-related disorders, and male infants have a higher risk of mortality than female infants.

## Introduction

The first year of life, or infancy, is considered to be a critical time for babies as—due to their developing organs and immune system—they are vulnerable to external environmental factors. The death of an infant before his or her first birthday is denoted as “infant mortality.” Infant mortality is usually interpreted and presented as the number of deaths per 1000 live births, or infant mortality rate (IMR) [1]. IMR is an important community health indicator for a geographical location or region and provides a fair estimate of population well-being. Infant mortality parameters could also indicate the extent and quality of healthcare services available to residents in a community [1].

Worldwide, according to the World Health Organization’s (WHO) estimates in 2015, 75% of all global deaths of children under five years were infants [2]. About 4.5 million deaths were attributed to infant mortality worldwide. Even though there has been a decline observed in annual infant deaths from 8.9 million in 1990 to 4.5 million in 2015 (63 to 32 deaths per 1000 live births), infant mortality is still considered a major financial and public health challenge worldwide [2].

The Arab world, along with the state of Qatar, has also experienced a decline in infant mortality [3]. According to the Qatar 2006 World Health Survey, records of vital statistics indicate that infant mortality decreased from 17 out of 1,000 live births per year in 1981 to 8.2 in 2006, an almost 50% drop in infant mortality between the two years in Qatar [4]. This decline may be attributed to the expansion of health services and healthcare delivery systems that led to higher quality healthcare in the country. Infant mortality is considered to be lower in Qatar than in other countries in the Eastern Mediterranean region [5].

The current health system in Qatar focuses on improving the health of people and maintaining their well-being by implementing multipronged approaches, such as improved access to high-quality health programs, promoting preventive services, and conducting health awareness campaigns. Qatar's health strategy targets, as a priority, women’s health (so that pregnancy is healthy) and children’s and adolescent health. The strategy emphasizes the implementation of programs consisting of high-quality maternity care throughout all stages of pregnancy and the postpartum period, and baby-friendly programs, breastfeeding promotion initiatives, early nutrition guidance, and childhood vaccination [6].

Clinically, the leading causes of infant mortality are birth defects, low birth weight (LBW) and prematurity, complications of pregnancy, sudden infant death syndrome (SIDS), and injuries. Other contributory factors include infections, malnutrition, level and quality of healthcare access, race, ethnicity, parental/family behavior, educational and socioeconomic status, and geographical region [7-9]. In the United States alone, about 57% of infant mortality-related deaths in 2014 were due to the top five causes together [1].

This article attempts an overview of the causes, demographics, frequency, and occurrence of infant mortality in Qatar retrospectively from 2004 through 2014. Since the area has not been extensively explored and published on in the past, this article is an attempt to provide an overview of infant mortality in Qatar. Specifically, the article aims to describe the leading causes of infant-related mortality by available demographic categories along with historical and recent trends. This information could help with applying evidence-based strategies targeting maternal and child health (MCH) services in Qatar. Local and regional health professionals and policymakers may also find the information useful to understand the IMR situation in the area and the associated service needs.

## Materials and methods

Mortality data collection and management

Infant mortality data for 2004 to 2014 were retrospectively examined from the database maintained by the Ministry of Public Health (MOPH). The mortality data are primarily collected through three sources: hospitals, the Ministry of Interior, and trauma emergency departments; and is considered comprehensive. A notification of death writes the description of the cause of death. The notification is sent to the MOPH, who issues the death certificate. The MOPH codes the causes of death by the International Statistical Classification of Diseases and Related Health Problems (ICD-10, Version 2016). Data derived from this process become part of the birth and death registration system. The system contains information such as date, place, and cause of death, along with demographic information such as age, gender, and nationality of the deceased.

We analyzed data from the MOPH database on deaths among infants (age at death less than or equal to one year) in Qatar from 2004 to 2014. We excluded from our analysis abortive deaths and deaths among Qatari nationals or long-term residents (non-Qataris) who were not in Qatar at the time of death. Data were presented by age groups, nationality, and gender. Infant age was categorized as less than one week (early neonatal), between one week and 27 days (late neonatal), and between 28 days (>27 days) and one year (post-neonatal). Nationality was categorized into Qatari and non-Qatari. Causes of deaths were presented with ICD codes and categorized by nationality and gender.

The change in infant mortality was expressed as a percentage change in the IMR. For example, the percentage of change in IMR between 2004 and 2014 is calculated using the formula (IMR 2014 – IMR 2004 / IMR 2004) x 100.

Ethical procedures were followed throughout the study. Confidential data and records handling were practiced. The registry data is kept in password-protected MOPH computers and used without personal identifiers. 

Statistical analysis

Frequencies, percentages, and rates per 1,000 live births were used to describe mortality data by nationality, gender, and age group. Daily mortality data were analyzed using their mean and standard deviation. Bivariate analysis of relative risks (RR) of infant mortality was conducted by nationality and gender. The 95% confidence interval (CI) was calculated according to Daly (1998) and reported as suggested by Altman (1998) [10-11]. The statistical significance level considered was five percent. Time trend was assessed by using monthly and yearly aggregation of the original database for all infant mortality cases by nationality. We used the nonparametric test for trend across ordered groups developed by Cuzick (1985), which is an extension of the Wilcoxon rank-sum test. This analysis was carried out using Stata, version 13 (StataCorp LLC, College Station, TX) and Excel 2013 (Microsoft Corp, Redmond, WA). 

## Results

Table 1 summarizes counts, rates, and infant mortality-related population parameters. Between 2004 and 2014, 204,224 live births and 1,505 infant deaths occurred in Qatar. The IMR averaged 7.37 per 1000 live births. The average IMR was slightly lower among Qatari nationals (6.81 per 1000 live births) than among non-Qataris (7.73 per 1000 live births). IMR was higher among males (8.1 per 1000 live births) than among females (6.6 per 1000 live births). Out of all live births (n=204,224), about 39% infants were Qatari, 61% non-Qatari, 51% male, and 49% female.

Of the infants who died between 2004 and 2014 (n=1,505), 36% were Qatari, 64% non-Qatari, 56% were male, and 44% were female. The results show that infant deaths have increased (Table 1). In 2004, there were 112 infant deaths (Qatari 45, non-Qatari 67). In 2014, there were 174 infant deaths (Qatari 50, non-Qatari 124). The mortality count did not change much among Qatari infants but increased among non-Qatari infants.

**Table 1:** Total live births, number of deaths, infant mortality rate, and daily average of mortality by nationality and sex from 2004 to 2014 IMR: Infant mortality rate

		Overall	Nationality		Sex	
			Qatari	Non-Qatari	Male	Female
2004	Total live births	13190	6488	6702	6802	6388
	Number of deaths	112	45	67	62	50
	IMR per 1000 live births	8.49	6.94	10.00	9.11	7.83
	Mean of death per day (SD)	0.31 (0.55)	0.12 (0.33)	0.18 (0.42)	0.17 (0.42)	0.14 (0.36)
2005	Total live births	13401	6260	7141	6839	6562
	Number of deaths	110	48	62	60	50
	IMR per 1000 live births	8.21	7.67	8.68	8.77	7.62
	Mean of death per day (SD)	0.30 (0.59)	0.13 (0.38)	0.17 (0.41)	0.16 (0.41)	0.14 (0.39)
2006	Total live births	14120	6563	7557	7196	6924
	Number of deaths	121	58	63	69	52
	IMR per 1000 live births	8.57	8.84	8.34	9.59	7.51
	Mean of death per day (SD)	0.33 (0.60)	0.16 (0.39)	0.17 (0.43)	0.19 (0.45)	0.14 (0.39)
2007	Total live births	15681	7178	8503	8056	7625
	Number of deaths	115	47	68	58	57
	IMR per 1000 live births	7.33	6.55	8.00	7.20	7.48
	Mean of death per day (SD)	0.32 (0.57)	0.13 (0.38)	0.19 (0.42)	0.16 (0.39)	0.16 (0.41)
2008	Total live births	17658	7660	9998	8927	8731
	Number of deaths	135	41	94	76	59
	IMR per 1000 live births	7.65	5.35	9.40	8.51	6.76
	Mean of death per day (SD)	0.37 (0.61)	0.11 (0.33)	0.26 (0.51)	0.21 (0.46)	0.16 (0.41)
2009	Total live births	18572	7523	11049	9496	9076
	Number of deaths	129	51	78	72	57
	IMR per 1000 live births	6.95	6.78	7.06	7.58	6.28
	Mean of death per day (SD)	0.35 (0.60)	0.14 (0.38)	0.21 (0.47)	0.20 (0.46)	0.16 (0.39)
2010	Total live births	19504	7733	11771	9926	9578
	Number of deaths	138	53	85	70	68
	IMR per 1000 live births	7.08	6.85	7.22	7.05	7.10
	Mean of death per day (SD)	0.38 (0.65)	0.15 (0.40)	0.23 (0.51)	0.19 (0.46)	0.19 (0.42)
2011	Total live births	20762	7687	13075	10551	10211
	Number of deaths	157	51	106	89	68
	IMR per 1000 live births	7.56	6.63	8.11	8.44	6.66
	Mean of death per day (SD)	0.43 (0.69)	0.14 (0.36)	0.29 (0.60)	0.24 (0.50)	0.19 (0.47)
2012	Total Live births	21819	7333	14486	11082	10735
	Number of deaths	151	48	103	95	56
	IMR per 1000 live births	6.92	6.55	7.11	8.57	5.22
	Mean of death per day (SD)	0.41 (0.67)	0.13 (0.35)	0.28 (0.54)	0.26 (0.55)	0.15 (0.39)
2013	Total live births	23890	7894	15996	12206	11684
	Number of deaths	163	55	108	101	62
	IMR per 1000 live births	6.82	6.97	6.75	8.27	5.31
	Mean of death per day (SD)	0.45 (0.64)	0.15 (0.39)	0.30 (0.52)	0.28 (0.51)	0.17 (0.39)
2014	Total live births	25627	8042	17585	13067	12560
	Number of deaths	174	50	124	93	81
	IMR per 1000 live births	6.79	6.22	7.05	7.12	6.45
	Mean of death per day (SD)	0.48 (0.74)	0.14 (0.40)	0.34 (0.60)	0.25 (0.48)	0.22 (0.48)
2004-2014	Total live births	204224	80361	123863	104148	100074
	Number of deaths	1505	547	958	845	660
	IMR per 1000 live births	7.37	6.81	7.73	8.11	6.60
	Mean of death per day (SD)	0.37 (0.63)	0.14 (0.37)	0.24 (0.50)	0.21 (0.47)	0.16 (0.41)

**Figure 1:**
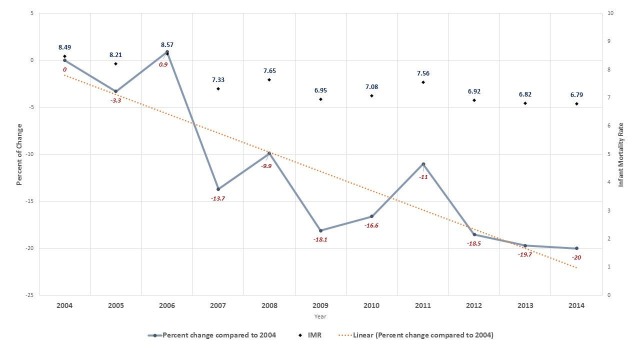
Percent change in infant mortality in Qatar from 2004 to 2014

The Ministry of Development Planning and Statistics estimates that the population of the State of Qatar increased from 744,029 in 2004 to 2,216,180 in 2014 (~197% increase). This increase is attributed mainly to the rapid in-migration of foreign workers in the past 10 years. Between 2004 and 2014, live births among non-Qataris (n=123,863) exceeded those among Qataris (n=80,361). Overall, the number increased from 13,190 in 2004 to 25,627 in 2014 (Table 1). However, the rates have decreased gradually: IMR declined 20% over 2004–2014 (Table 1 and Figure 1). Mortality rates were higher among male infants than among female infants in all years except in 2007 (Table 1).

**Figure 2:**
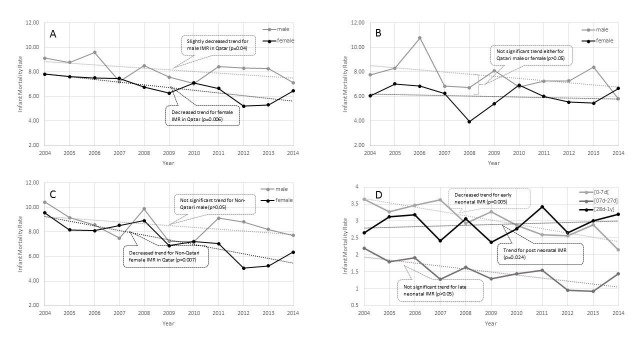
Infant mortality rate by gender; A: for all population, B: for Qatari, and C for non-Qatari. D: Infant mortality rate by age groups for the whole population

The percentage decline in IMR was not statistically significant among Qatari nationals (overall p=0.15, males p=0.27, females p=0.35), but significant among the overall non-Qatari population (p=0.007) and females only (p=0.007) (Figure 2). A decreasing trend was observed for early and late-neonates, but the trend for late-neonates was not statistically significant. A fluctuation in IMR was observed for post-neonatal births, with a slightly increasing trend (p=0.02) (Figure 2). The decrease in IMR from 2004 to 2014 was statistically significant for the overall population (p=0.006), males (p=0.04), and females (p=0.006) (Figure 2).

The most common causes of infant deaths were disorders related to short gestation and LBW (n=400, 27%). Other causes were congenital heart malformations (n= 137, 9%), congenital malformations of lungs (n=89, 6%), other congenital malformations (n=47, 3%), and respiratory distress (n=43, 3%). A total of 520 infants (35%) died because of congenital malformations, deformation, and chromosomal disorders (Q00-Q99), which come under ICD Chapter 17. Only 19 cases of infant mortality between 2004 and 2014 were due to R95 or SIDS; no case was reported in 2008, 2012, or 2013. SIDS-related deaths numbered 1–3 per year for the rest of the period, except 2005 (when the count was six). Only 18 deaths were associated with infectious diseases (ICD, Chapter 1); 13 of these were attributed to sepsis (Other).

The RR for the top causes of infant deaths are shown in Table 2. The results reveal that in the overall population, male infants had a statistically significant higher risk of mortality than female infants due to the congenital malformation of lungs (RR=1.63, CI= 1.06–2.51, p=0.0259), other congenital malformations (RR=2.26 CI=1.21–4.23, p=0.0104), and cardiovascular disorders (RR=2.28 CI=1.00–5.21, p=0.05) (Table 2). Qatari male infants had a twofold higher risk of mortality (2.02 times) than female infants due to the congenital malformation of lungs (CI=1.02–4.03, p=0.0447). Non-Qatari male infants had higher risks of mortality than non-Qatari female infants due to other congenital malformation (RR=2.33, CI=1.07–5.07, p=0.0323) and cardiovascular disorders (RR=3.82, CI=1.28–11.42, p=0.02) (Table 2).

**Table 2:** Top causes of infant mortality from 2004-2014 (overall) by nationality and gender IMR: Infant mortality rate

Cause of Death (ICD 10 Codes)	Total Number of Deaths(Selected top causes)	% of Total Infant Mortality (N=1505)	Qatari IMR (n)	Non-Qatari IMR (n)	RR (95% CI)	Qatari Male IMR (n)	Qatari Female IMR (n)	RR (95% CI)	Non-Qatari Male IMR (n)	Non-Qatari Female IMR (n)	RR (95% CI)	Male IMR (n)	Female IMR (n)	RR (95% CI)	
Congenital Malformations All* (Q00-Q99)	520	34.5	2.44 (196)	2.62 (324)	0.93 (0.78 - 1.11)	2.72 (111)	2.15 (85)	1.27 (0.96 - 1.68)	2.75 (174)	2.48 (150)	1.11 (0.89 - 1.38)	2.74 (285)	2.35 (235)	1.17 (0.98 - 1.38)	
Low Birth Weight (P07)	400	26.6	2.00 (161)	1.93 (239)	1.04 (0.85 - 1.27)	2.48 (101)	1.52 (60)	1.27 (0.96 - 1.68)	2.00 (127)	1.85 (112)	1.11 (0.89 - 1.38)	2.19 (228)	1.72 (172)	1.17 (0.98 - 1.38)	
Congenital Malformations of Heart (Q24)	137	9.1	0.57 (46)	0.73 (91)	0.78 (0.55 - 1.11)	0.47 (19)	0.68 (27)	0.68 (0.38 - 1.23)	0.71 (45)	0.76 (46)	0.93 (0.62 - 1.41)	0.61 (64)	0.73 (73)	0.84 (0.60 - 1.18)	
Congenital Malformations of Lungs (Q33)	89	6.0	0.46 (37)	0.42 (52)	1.10 (0.72 - 1.67)	0.61 (25)	0.30 (12)	2.02 (1.02 - 4.03)ⱡ	0.49 (31)	0.35 (21)	1.41 (0.81 - 2.45)	0.54 (56)	0.33 (33)	1.63 (1.06 - 2.51)ⱡ	
Other Congenital Malformations (Q89)	47	3.1	0.20 (16)	0.25 (31)	0.80 (0.44 - 1.45)	0.27 (11)	0.13 (5)	2.14 (0.74 - 6.15)	0.35 (22)	0.15 (9)	2.33 (1.07 - 5.07)ⱡ	0.32 (33)	0.14 (14)	2.26 (1.21 - 4.23)ⱡ	
Respiratory Distress of Newborn (P22)	43	2.8	0.22 (18)	0.20 (25)	1.11 (0.61 - 2.03)	0.29 (12)	0.15 (6)	1.94 (0.73 - 5.18)	0.17 (11)	0.23 (14)	0.75 (0.34 - 1.65)	0.22 (23)	0.20 (20)	1.11 (0.61 - 2.01)	
Edwards & Patau Syndrome (Q91)	30	2.0	0.09 (7)	0.19 (23)	0.47 (0.20 - 1.09)	0.10 (4)	0.08 (3)	1.30 (0.29 - 5.79)	0.16 (10)	0.21 (13)	0.73 (0.32 - 1.67)	0.13 (14)	0.16 (16)	0.84 (0.41 - 1.72)	
Cardiovascular Disorders (P29)	27	1.8	0.09 (7)	0.16 (20)	0.54 (0.23 - 1.28)	0.07 (3)	0.10 (4)	0.73 (0.16 - 3.25)	0.25 (16)	0.07 (4)	3.82 (1.28 - 11.42)ⱡ	0.18 (19)	0.08 (8)	2.28 (1.00 - 5.21)ⱡ	
* Congenital malformations, deformations and chromosomal abnormalities(complete ICD-10 Chapter 17) also includes Q24, Q89, Q91 listed separately in the table ⱡ Statistically significant with a p value ≤0.05															

**Figure 3:**
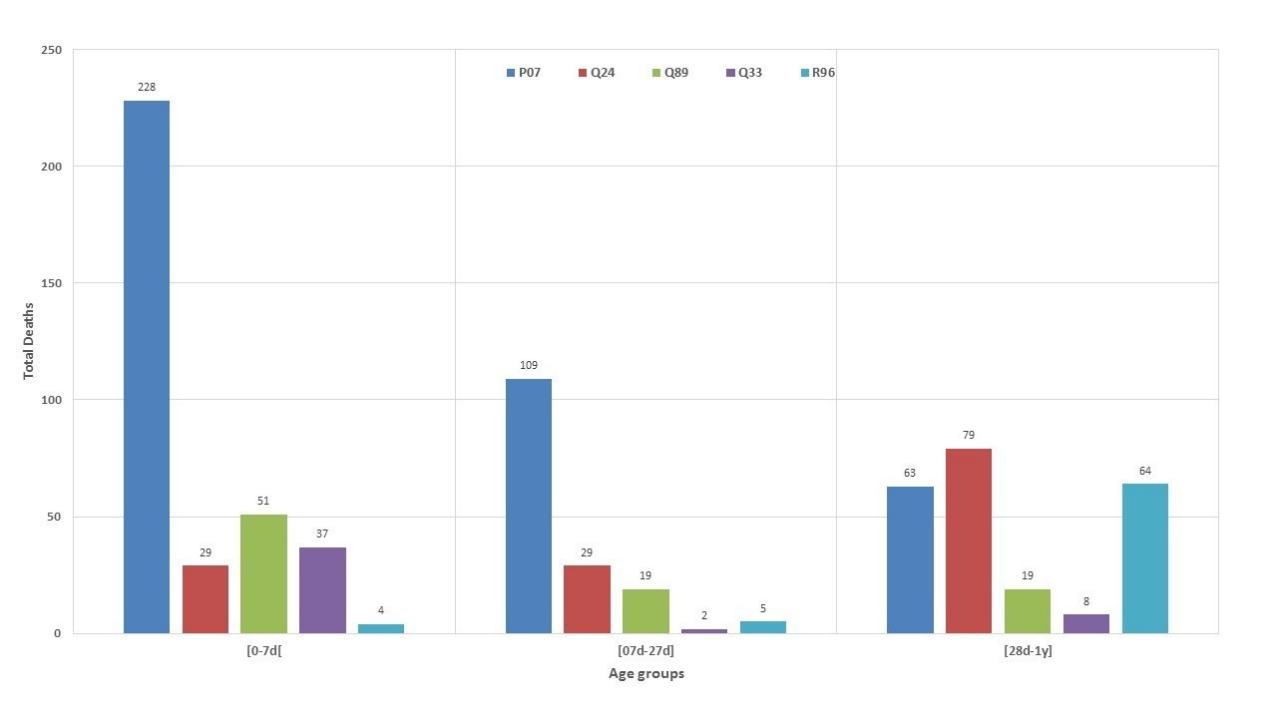
Top causes of infant mortality by age groups P07: Low birth weight Q24: Congenital malformations of heart (other) Q89: Congenital malformations (other) Q33: Congenital malformations of lungs R96: Other sudden death, cause unknown

Most common causes of infant mortality by age groups are shown in Figure 3. Low birth weight related deaths were highest during early neonatal (n=228) and late neonatal (109) period. Other-congenital malformations of heart-related deaths had the highest count among post-natal infants (Figure 3).

**Figure 4:**
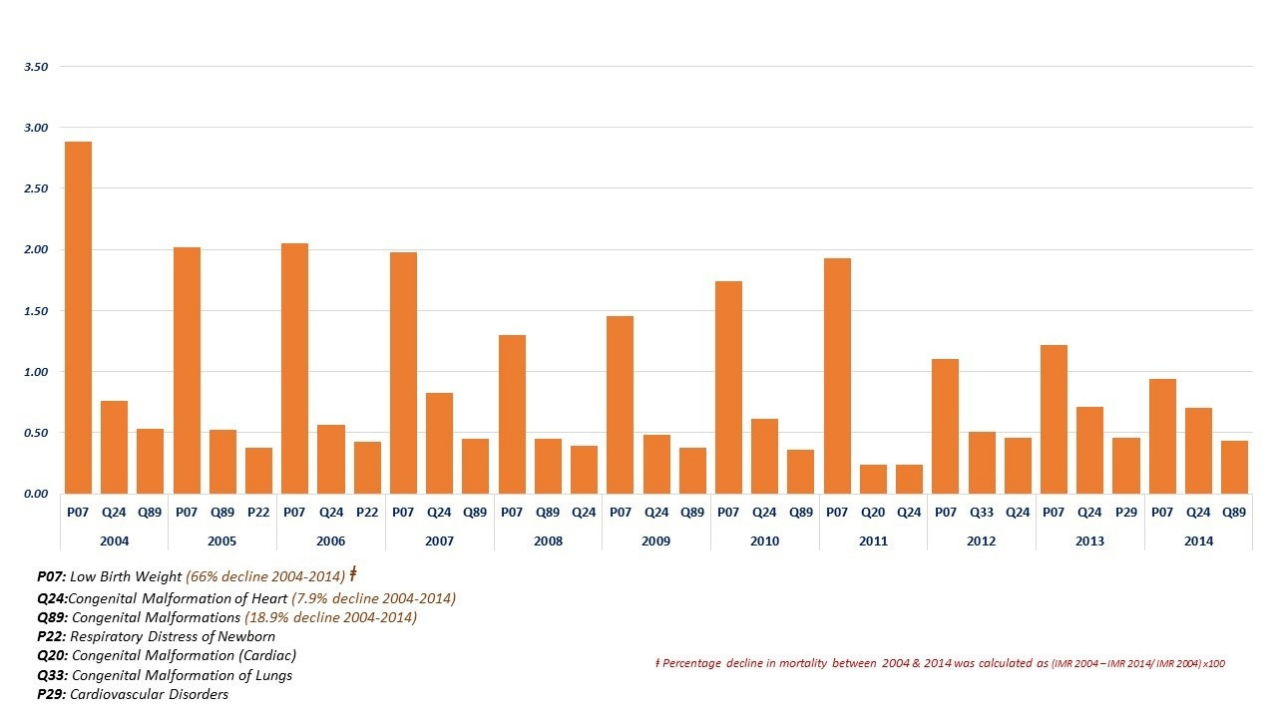
Top three causes of infant mortality in each year, Qatar 2004-2014

The top three causes of infant mortality by year are shown in Figure 4. Deaths related to birth weight were higher than other causes between 2004 and 2014; however, mortality due to LBW declined 66%. There was a decline also in other congenital malformations (19%) and congenital malformations of the heart (8%).

## Discussion

This article describes retrospective infant mortality data in Qatar. The population of Qatar has changed, mainly due to migrant workers. This change has led to a gradual increase in the number of live births among non-Qataris and the total population. The number of births among Qataris has been relatively stable during the period (Table 1). The population dynamics in Qatar are unique due to the high number of migrant workers; the results show a downward pattern in IMR, which can be expected in countries with recent socioeconomic development [12]. The WHO estimates that between 1990 and 2013, IMR declined 55.4% in high-income countries and 46.4% worldwide. In 1990, the IMR in Qatar was 17.7 per 1,000 live births, which brings down the percentage of IMR decline to 60.5% in 2013. This drop is similar to that in neighboring Saudi Arabia (60%), higher than in the United Arab Emirates (51%) and Kuwait (43.7%), and lower than in Oman (62.3%) and Bahrain (73.3%) [13]. Interestingly, between 2000 and 2013, the percentage of decline in IMR was similar in high-income countries (34.6%), worldwide (36.6%), and in Qatar (34.6%) [13].

Indicator 4.2, which targets infant mortality, was one of the components of Millennium Development Goal (MDG) goal number 4 aiming to reduce the mortality rates among children under five years by two-thirds between 1990 and 2015 [14]. If we consider only IMR, two-thirds of the IMR in Qatar in 1990 (17.7 per 1,000 live births) is 11.8. Qatar’s IMR In 2014, 6.91 per 1,000 live births, was better than the fourth MDG, but higher than the Healthy People 2020 goal of 6.0 per 1,000 live births [15]. The fourth MDG was to reduce the mortality rate among children under five years by two-thirds between 1990 and 2015

Our article shows that on average and across all years (except 2007), the IMR was higher among male infants than among female infants. This is consistent with the findings of an Indian retrospective study published in 2007 [8]. A study in Kuwait showed, similar to our findings, that mortality was higher among male infants than among female infants and that infant mortality was higher among expatriates or foreign residents than among local nationals [16].

In Qatar, the major causes of infant mortality are congenital malformations of all types and LBW, including prematurity. These findings are consistent with those of the Centers for Disease Control and Prevention (CDC) and of a Saudi study [1,17]. It is evident from other studies that genetic and congenital disorders, and the associated infant morbidity and mortality, are higher among Arab nations than among other industrialized countries [18-19]. Other main causes of mortality of newborns are respiratory distress syndrome and cardiovascular disorders. The data showed that male infants have a higher risk of dying from certain diseases and conditions, such as congenital malformations of other types and lung and cardiovascular disorders. This finding could support the fact that male infants are more likely than female infants to die in their first year of life [20-21].

Various programs have been planned and implemented to promote community health. Many of these target MCH. From 2012 to 2014, the number of physicians grew by 35%, and the total healthcare and -related workforce increased to 23% [22]. In addition to the advanced health care system, new antenatal care models focusing on health education pertaining to MCH have been implemented in Qatar [22].

The article represents a favorable situation in the form of reducing IMR in Qatar. These findings could be useful in strengthening the MCH initiatives in the country. The results could also be useful for other nations, especially those within the Gulf Cooperation Council, which experience population circumstances that are similar to those of Qatar. The study urges others to incorporate evidence-based best practices related to health promotion and education in existing programs. Specifically, practices such as breastfeeding, folic acid use during pregnancy, prenatal and inter-conception care, and controlling chronic diseases could be very beneficial in reducing poor birth outcomes [23]. For example, optimal periodontal care is associated with lower risks for low-weight/preterm births, and since periodontal health has been identified as a challenge in children and adults–especially among females residing in Qatar–the existing programs could require modification to address multiple outcomes, and further studies are needed to examine this relationship [23-24]. The relationship between obesity and IMR is well known; given the high obesity among the female population in Qatar, public health programs should incorporate multidimensional approaches to monitor infant mortality [25-27].

The strength of this article is that there is little chance that data are missing, as the data were obtained from the birth and death registry maintained by the MOPH, the leading agency that handles these registrations in the State of Qatar, and, as a result, the study represents all deaths that occurred in Qatar during 2004–2014. One limitation of this data is that, sometimes, the final diagnosis or circumstances associated with the death reported in the notification are unclear and could cause difficulties in ICD coding. Furthermore, the population of Qatari nationals is relatively stable compared to that of non-Qataris. This has an impact on several key health indicators, for example, the death rates in Qatar could be influenced by the large proportion of relatively young healthy migrants added to the population. This type of population situation creates a selection bias, especially in mortality indicators.

## Conclusions

In Qatar, mortality is higher among male infants than among female infants, as is the risk of mortality, due to the most common causes of infant mortality. The results of this study could play an important role in the introduction of customized strategies and policies to address identified issues.
